# Synthesis and Characterization of Catalytically Active Au Core—Pd Shell Nanoparticles Supported on Alumina

**DOI:** 10.1021/acs.langmuir.2c01834

**Published:** 2022-10-12

**Authors:** Yanyue Feng, Andreas Schaefer, Anders Hellman, Mengqiao Di, Hanna Härelind, Matthias Bauer, Per-Anders Carlsson

**Affiliations:** † Department of Chemistry and Chemical Engineering, Chalmers University of Technology, SE-412 96Gothenburg, Sweden; ‡ Department of Physics, Chalmers University of Technology, SE-412 96Gothenburg, Sweden; § Department of Chemistry, Paderborn University, 33098Paderborn, Germany

## Abstract

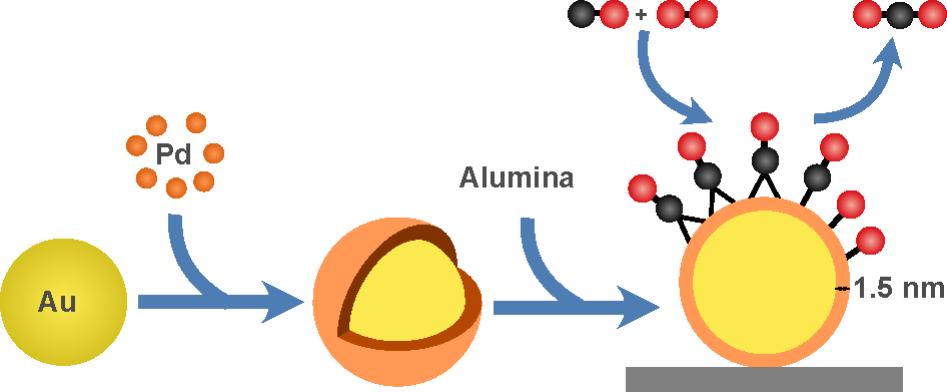

A two-step seeded-growth method was refined to synthesize Au@Pd core@shell nanoparticles with thin Pd shells, which were then deposited onto alumina to obtain a supported Au@Pd/Al_2_O_3_ catalyst active for prototypical CO oxidation. By the strict control of temperature and Pd/Au molar ratio and the use of l-ascorbic acid for making both Au cores and Pd shells, a 1.5 nm Pd layer is formed around the Au core, as evidenced by transmission electron microscopy and energy-dispersive spectroscopy. The core@shell structure and the Pd shell remain intact upon deposition onto alumina and after being used for CO oxidation, as revealed by additional X-ray diffraction and X-ray photoemission spectroscopy before and after the reaction. The Pd shell surface was characterized with in situ infrared (IR) spectroscopy using CO as a chemical probe during CO adsorption–desorption. The IR bands for CO ad-species on the Pd shell suggest that the shell exposes mostly low-index surfaces, likely Pd(111) as the majority facet. Generally, the IR bands are blue-shifted as compared to conventional Pd/alumina catalysts, which may be due to the different support materials for Pd, Au versus Al_2_O_3_, and/or less strain of the Pd shell. Frequencies obtained from density functional calculations suggest the latter to be significant. Further, the catalytic CO oxidation ignition-extinction processes were followed by in situ IR, which shows the common CO poisoning and kinetic behavior associated with competitive adsorption of CO and O_2_ that is typically observed for noble metal catalysts.

## Introduction

The transformation of one-carbon compounds (C 1s) is important for the synthesis of liquid fuels and chemical building blocks and plays a crucial role in environmental protection. Common C 1s include methane (CH_4_), methanol (CH_3_OH), formic and carbonic acids (HCOOH and HOCOOH), and oxides (CO and CO_2_). Both CO_2_ and CH_4_ are naturally abundant. Hitherto, the latter is a more easily processed feedstock^1^ that can be obtained from renewable sources, which is of utmost importance for the transition to sustainable chemical production.^2−5^ Concerning emission abatement, the complete oxidations of poisonous CO and CH_4_, the second most important greenhouse gas, are important reactions to improve. In this connection, the design of model and applicable materials exposing surfaces that possess catalytic activity for the desired reaction(s) is crucial.

For catalytic oxidation of both CO and CH_4_, palladium is often the element of choice when designing the active component of the catalyst.^6−9^ Under oxidizing conditions at low temperatures, Pd-based catalysts typically undergo oxidation such that PdO develops, which becomes the active component in practice.^10−15^ For ideal single crystals, it has been shown that a thin layer of PdO(101) can form on the PdO(100) surface when exposed to oxygen. The PdO(101) surface exposes under-coordinated Pd atoms that excel the catalytic activity for both CO^16,17^ and CH_4_ oxidation.^18,19^ If the PdO layer grows too thick, a nonepitaxial growth takes over such that a polycrystalline PdO develops, which has a low catalytic activity.^20^ Hence, it is of great interest to explore palladium-based catalysts for which thick PdO layers are avoided by design, that is, for which the possible development of PdO is limited to a handful of PdO layers at the maximum, without losing beneficial catalytic properties. Further, as the specific active surface area is one key performance parameter for heterogeneous catalysts, suitable catalysts cannot be based on ideal surfaces but need to rely on small particles to provide the necessary specific number of active sites. Practically, the particles are dispersed onto a support material that carries and stabilizes the particles and prevents them from sintering when exposed to reaction or other harsh conditions. In this respect, alumina is a suitable support for palladium and is commonly used in technical catalysts. This is despite the fact that the palladium–alumina system is sensitive to water that forms site-blocking surface hydroxyls.^21−23^ It is thus essential to find catalyst designs in which the palladium is separated from the alumina, that is, supported onto another material.

Catalyst synthesis methods are continuously advanced, and during the last decades, synthesis routes allowing for the preparation of various bimetallic catalysts have been developed. The main idea is that the flexibility of the design of the bimetallic phase allows steering structural properties of importance for the catalytic action. There are numerous bimetallic systems, and among these, a few Au–Pd systems have been reported to offer superior catalytic performance compared with their palladium monometallic counterparts.^9,24,25^ A common focus has been on Au–Pd alloys for which enhanced catalytic activity has been ascribed to both ensemble and ligand effects as a result of the two interacting elements.^25^ Likewise, the distribution of the included atoms affects the catalytic activity.^26,27^ Another architecture of bimetallic systems that is relevant here is the core–shell motif with a Au core surrounded by a Pd shell, also denoted as the Au@Pd core@shell structure. Such systems have been proposed to be of particular interest for catalysis not only because of ligand effects but also from the fact that the Pd surface atoms may experience strain and the Au core can modify their electronic structure.^26,28−32^ The Au@Pd core@shell motif, even when supported on an alumina, provides opportunities to tackle both catalytic challenges discussed above as the Pd thickness can be limited to a few atom layers and the contact between Pd and alumina minimized.

Here, we report on the development of a wet-chemical synthesis concept for the preparation of Au@Pd core@shell nanoparticles with thin shells and the subsequent deposition of these onto a common Al_2_O_3_ support as to make an applicable Au@Pd/Al_2_O_3_ catalyst design. The nanoparticles and especially those on the finished catalyst have been characterized using several methods including transmission electron microscopy (TEM) with energy-dispersive spectroscopy (EDS), X-ray diffraction (XRD), and X-ray photoelectron spectroscopy (XPS). Further, in situ infrared (IR) spectroscopy was used to study the surface Pd atoms using CO as a chemical probe molecule and the CO oxidation ignition–extinction processes. The measured frequencies are compared with previous reports, and the observed frequency shifts are compared also with those obtained from density functional calculations.

## Materials and Methods

### Chemicals, Precursors, and Gasses

The chemicals that were used in the synthesis include gold chloride trihydrate (HAuCl_4_·3H_2_O, Fisher), l-ascorbic acid (Fisher), palladium chloride (≥99%, Sigma-Aldrich), potassium carbonate (ACS reagent, ≥99%, Sigma-Aldrich), trisodium citrate (≥98.5%, VWR), and γ-alumina (Alfa Aesar). All chemicals were used as received. All stock and reaction solutions were prepared by Milli-*Q* water. HAuCl_4_ reaction solution (25 mM) was prepared by adding 197 mg of HAuCl_4_·3H_2_O in 20 mL of Milli-*Q* water. Besides, 10 mM H_2_PdCl_4_ solution was prepared by dissolving 17.7 mg of PdCl_2_ in 10 mL of 0.2 M HCl solution and then diluting to 100 mL.^33^ All glassware were cleaned with aqua regia prior to usage. The gases used for in situ spectroscopy were O_2_ (≥99.9992%), H_2_ (25%), and CO (2%) in Ar. Ar (99.998%) was used as the carrier gas.

### Synthesis of Au@Pd Core@Shell Nanoparticles

The core@shell Au@Pd nanoparticles were synthesized by a seeded-growth method, which is based on the method used by Piella et al.^34^ and Hu et al.,^35^ although with a few modifications. First, Au core nanoparticles were synthesized by reduction of HAuCl_4_ followed by nucleation and particle growth. 3 mL of 0.1 M l-ascorbic acid was mixed with 150 mL of 2.2 mM trisodium citrate solution and 1 mL of K_2_CO_3_ under vigorous stirring in a round-bottom flask and continuous heating. At the temperature of 70 °C, 1 mL of 2.5 mM HAuCl_4_ solution was added dropwise. The color of the solution changed immediately to pink-red, indicating the formation of Au nanoparticles, and the reaction was kept for 10 min. The as-prepared Au nanoparticles were used as the core material upon which a Pd shell was grown. To obtain a thin Pd shell, around 1.5 nm, 50 mL of the solution containing the Au nanoparticles was mixed with 0.25 mL of 10 mM H_2_PdCl_4_ solution. Both solutions were cooled to 1 °C before mixing. Then, 2.5 mL of ice-cold 0.1 M l-ascorbic acid was added dropwise while stirring. The obtained solutions containing the Au@Pd core@shell nanoparticles were dark brown.

### Synthesis of the Au@Pd/Al_2_O_3_ Catalyst

The solutions containing the Au@Pd core@shell nanoparticles were concentrated by centrifugation before loading onto the alumina support. The supernatant was removed to eliminate the small nanoparticles as well as the free precursor ions. For the loading procedure, 0.4 g of γ-Al_2_O_3_ powder was mixed with solutions with concentrated as-prepared Au@Pd core@shell nanoparticles and stirred overnight at room temperature. The obtained slurries were centrifuged under 5000 rpm and then freeze-dried.^36^

### Ex Situ Characterizations

The morphology of Au@Pd core@shell nanoparticles and the Au@Pd/Al_2_O_3_ catalyst were imaged with electron microscopy using an FEI Tecnai T20 microscope operated with an acceleration voltage of 200 kV in transmission mode TEM as well as an FEI Titan 80–300 microscope operated at 300 kV in the scanning mode scanning TEM (STEM). In addition, high-angle annular dark field (HAADF)–STEM and EDS line scanning were used to further probe the core@shell structure. To prepare the specimen, a droplet of as-prepared Au@Pd nanoparticles was loaded on a holey carbon film supported on a copper grid. As for Au@Pd/Al_2_O_3_, a small amount of sample was dispersed in ethanol. Then, the obtained suspension was drop-casted onto the holey carbon film. Moreover, the formation of Au nanoparticles and the growth of the Pd shell were also determined by an ultraviolet–visible spectrophotometer (UV–vis, NanoDrop One) in the wavelength range from 200 to 850 nm.

The crystal structure and crystal structure stability were investigated by XRD through a powder X-ray diffractometer using a D8 Advance diffractometer (Bruker) accompanied by Cu Kα as the X-ray source and a Lynx-eye energy dispersive detector. The XRD patterns were recorded in the 2θ value ranging from 20 to 90° under an ambient pressure with 0.02° incremental step and 1.8 s dwell time for each step. The sample preparation included loading Au@Pd/Al_2_O_3_ onto a capillary sample holder and flattening the surface with a glass slide. The contents of Pd and Au in the sample were measured by X-ray fluorescence (XRF) with an Axios spectrometer (Malven-Panalytical) housing a Rh anode. The samples were measured as loose powders in the He atmosphere in a polypropylene (PP) sample cup with a 6 mm PP film (ChemPlex). The quantification was based on calibration with Omnian setup samples (Malvern-Panalytical) and using the SuperQ software provided with the instrument.

### In Situ Characterizations

The adsorption of CO on the sample surface was analyzed by in situ diffuse reflectance IR Fourier transform spectroscopy (DRIFTS) using a VERTEX 70 FTIR spectrometer (Bruker). A high-temperature stainless steel reaction chamber (Harrick Inc.) with CaF_2_ window and a liquid nitrogen-cooled MCT detector were used. A water-cooling jacket was incorporated to control the temperature of the outer surface of both the reaction chamber and its windows. The temperature underneath the sample holder and in the top of the sample bed were measured by two type K thermocouples. The latter is hereafter referred to as the sample temperature. The desired feed gas composition was achieved by using individual mass flow controllers (Bronkhorst) for the mixing of gases. Ar (99.998%) was used as the carrier gas. The CO adsorption measurements were performed with only Au@Pd/Al_2_O_3_ powder in the sample bed, that is, no mixing with inert material such as KBr. The sample was pretreated at 198 °C with 3 vol % O_2_ for 20 min and then 3 vol % H_2_ for 1 h using a total constant gas flow of 100 mL/min. Upon exposure to 0.2 vol % CO, spectra ranging from 400 to 4000 cm^–1^ with a resolution of 1 cm^–1^ were collected under step-wise decreased temperature ranging from 198 to 33 °C, for which individual reference spectra in the presence of H_2_ had been recorded.

The oxidation of 0.2 vol % CO in excess O_2_ over the Au@Pd/Al_2_O_3_ powder was studied by in situ DRIFTS subsequently after the CO adsorption measurements in the same experimental setup. Again, spectra ranging from 400 to 4000 cm^–1^ with a resolution of 1 cm^–1^ were recorded for CO oxidation first for the ignition process for which the temperature was increased from 33 to 198 °C and then for the extinction process when the temperature was decreased back to 33 °C. The effluent gas flow downstream of the DRIFTS cell was continuously analyzed with a mass spectrometer (Hiden Analytical) following the *m*/*z* 28 (CO), 32 (O_2_), 40 (Ar), and 44 (CO_2_).

### Density Functional Calculations

Density functional calculations were performed using VASP^37−39^ with the PBE exchange–correlation functional.^40^ The projector augmented wave method^41^ was used to model the interaction between the valence electrons and the core. The Kohn–Sham orbitals were represented using a plane-wave basis set with 450 eV as cutoff energy, and a Gaussian smearing of 0.05 eV was applied to the Fermi level discontinuity. The Pd(111) surface was modeled as a five-layer *p*(2 × 2) supercell. The periodic slabs were separated by 16 Å. The Brillouin zone was sampled using the Monkhorst–Pack grid^42^ with (8 × 8 × 1) *k*-points. The gas-phase CO was treated in a cubic box with sides of 10 Å. In order to systematically study the different adsorption sites on Pd(111), that is, ontop, bridge, hcp, and fcc sites, the adsorbates were constrained to the specific sites; however, no other constraints were enforced. The ionic positions were considered to be relaxed when the largest atomic force in the system was smaller than 0.01 eV/Å. Vibrational energies were calculated by constructing the Hessian matrix using atomic forces generated by 0.01 Å displacements of the considered atoms.

## Results and Discussion

### Synthesis Strategy

**Figure 1:**
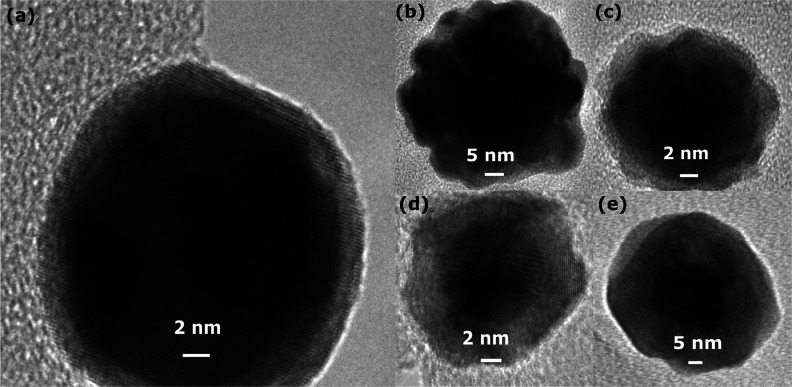
Effect of temperature and Pd/Au molar ratio on the morphology of Au@Pd nanoparticles. (a) 1 °C, Pd/Au = 0.31; (b) 80 °C, Pd/Au = 0.31; (c) 20 °C, Pd/Au = 0.31; (d) 1 °C, Pd/Au = 3.1; (e) 1 °C, Pd/Au = 31.

Previous studies have outlined two mechanisms for the growth of Pd shells on Au cores, viz., nuclei coalescence and monomer attachment.^43^ Whether the growth follows one or the other mechanism, or a combination of the two, differs among systems. Aiming at creating particles with well-controlled Pd shells, one may envisage that it is desirable to target the Pd monomer attachment route to achieve thin and even Pd shells instead of the nuclei coalescence route that hypothetically may result in thicker and rougher Pd shells. However, independent of the formation mechanism, which is challenging to reveal experimentally, it has been shown that the morphology of the core@shell nanoparticles depends on several operational parameters during synthesis, such as the reaction temperature^43^ and the type and ratio of synthesis precursors.^44^ The reaction temperature determines whether the growth is under thermodynamic or kinetic control,^45^ whereas the Pd/Au molar ratio influences the shell thickness. With the desire to influence the catalytic properties of the shell by affecting its structure, it is thus of great importance to balance the synthesis parameters so as to achieve a certain morphology of the core@shell nanoparticles.^44^ The present synthesis is based on previous methods^34,35^ but refined to use l-ascorbic acid instead of tannic acid as a reducing agent for the synthesis of the Au cores. Using l-ascorbic acid already when making the cores is advantageous for the subsequent synthesis of the Pd shell because it promotes the shell growth at the expense of the fast formation of Pd nanoparticles. This is because the mixing of tannic acid and l-ascorbic acid synergistically increases the reducing power of the acids leading to too fast reduction of the Pd precursor. Suppressing the amount of formed Pd nanoparticles favors the mechanism of monomer attachment, leading to a thin and even Pd shell. A drawback could be that the obtained Au nanoparticles are larger and present a broader size distribution as can be seen in Figure S1. This is, however, not of primary concern here as we target to develop the concept of synthesis of (clear) core–shell nanoparticles and support these onto alumina. Exploring the operational parameters, it turned out that keeping the temperature steady at 1 °C and using the Pd/Au ratio of 0.31 are critical for the growth of a thin and evenly distributed Pd shell on the Au cores as can be seen in the microscopic images in Figure 1a. This will be discussed more below. Using higher synthesis temperatures or Pd/Au ratios leads to thick and uneven shells and, in some cases, shells that appear to consist of agglomerated Pd particles as shown in Figure 1b–e.

**Figure 2:**
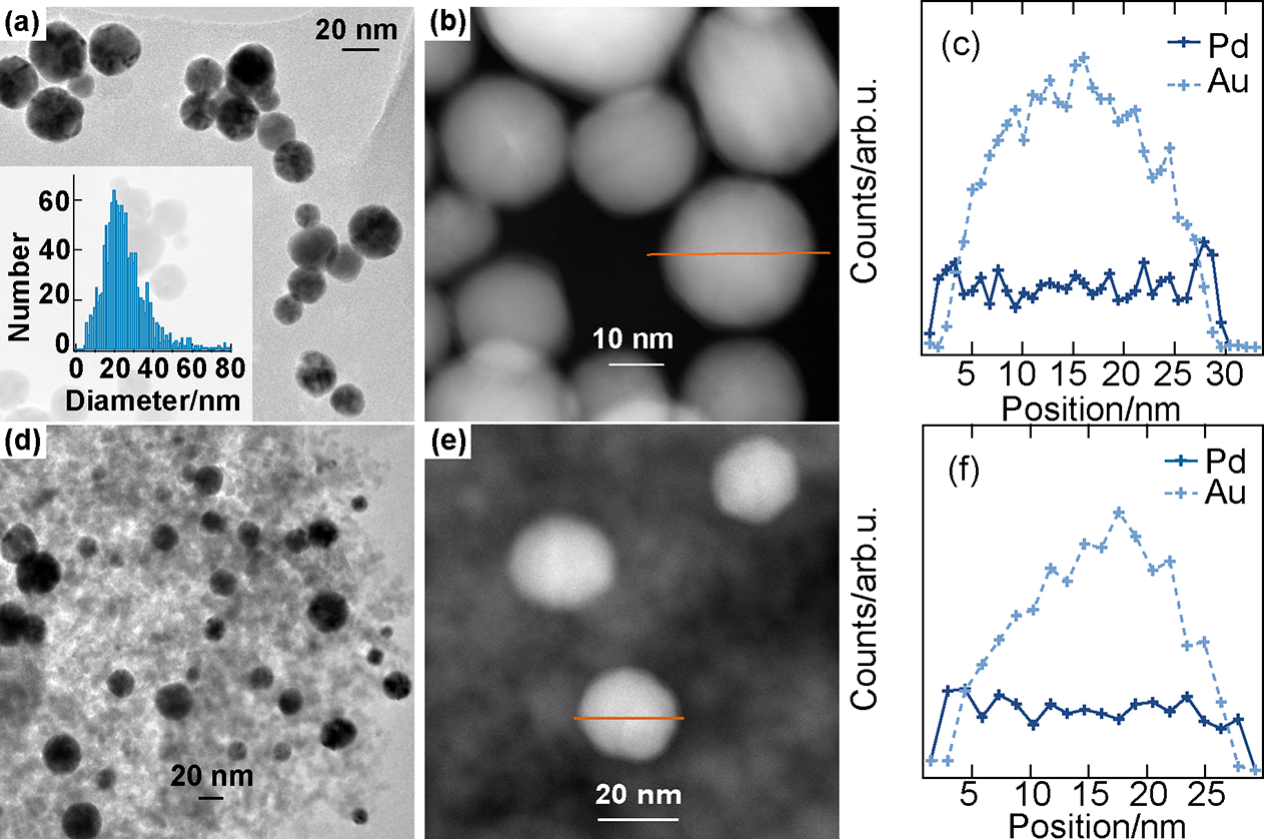
(a) TEM images of Au@Pd nanoparticles. (b) HAADF–STEM image of Au@Pd nanoparticles. (c) EDS line scan profile of Au@Pd nanoparticles. (d) TEM image of as-prepared Au@Pd/Al_2_O_3_ (e) HAADF–STEM image of as-prepared Au@Pd/Al_2_O_3_. (f) EDS line scan profile of as-prepared Au@Pd/Al_2_O_3_.

The use of nanoparticles for heterogeneous catalysis is seldom possible without carrying them on a suitable high-surface area support material that usually is in the form of a porous powder that can be practically handled. The synthesis of supported noble metal catalysts commonly relies on wet impregnation of the active element(s), in the form of a metal salt, onto the support, followed by drying and calcination. This leads to good contact between the impregnated element(s) and the support material, facilitating many catalytic properties, including robustness toward sintering. Unfortunately, the control of the size and composition of the nanoparticles is not straightforward. This motivates investigations on the deposition of prefabricated nanoparticles, with tailored size and composition, onto suitable supports.^46^ However, achieving sufficient anchoring of prefabricated nanoparticles to the support can be difficult due to weak interactions between nanoparticles and the support material. Here, we used a rather straightforward approach that includes concentration of as-prepared Au@Pd core@shell nanoparticles, direct mixing with alumina powder, and freeze-drying. Mixing is a key step in this sequence. As the as-prepared Au@Pd nanoparticles are stabilized by citrate ions, their (effective) surfaces are negatively charged, and as the inherent pH of 6.8 of the Au@Pd solution is kept unchanged, the alumina surface becomes positively charged upon mixing. Overall, this results in attractive interactions between the nanoparticles and the alumina, which allow for direct attachment of Au@Pd nanoparticles onto the alumina surface as can be seen in the microscopic images in Figure 2d,e.^47^ The resulting noble metal loading of the as-prepared Au@Pd/Al_2_O_3_ sample is in this case 0.009 w % Pd and 0.774 w % Au as measured by XRF.

### Morphology and Crystal Structure

The morphology of the core@shell nanoparticles alone and supported on alumina can be better understood by a detailed analysis of the TEM images presented in Figure 2. It is clear that the vast majority of the obtained Au@Pd core@shell nanoparticles are spherical and with a diameter ranging from 15 to 30 nm. The size distribution stems from the distribution of core sizes (cf. S1). A narrower core size distribution could possibly be achieved by using, for example, tannic acid as a reducing agent, during the synthesis of the Au cores. This reduces the Au(III) ions and stabilizes the nanoparticles. However, the concentration of tannic acid must be well controlled because excess amounts will lead to polydispersed nanoparticles.^34^ Nevertheless, the variation in the core size is not considered crucial here for the catalytic properties as a 15 nm Au particle still provides a large core, relative to the thin Pd shell, as a substrate for the palladium. Despite the size distribution, the Pd shell thickness of all Au@Pd nanoparticles is close to 1.5 nm, as shown by both the HAADF–STEM image and the EDS line scan profile in Figure 2b,c, respectively. Further, Figure S2 shows the thickness distribution of the Pd shells, which clearly is narrow. The thin shell consists of about five layers of palladium. Moreover, the growth of Pd shell onto Au nanoparticles was also characterized by UV–vis spectroscopy. The UV–vis absorption spectrum of as prepared Au and Au@Pd nanoparticles is shown in Figure S3. The Au nanoparticles exhibit an intense surface plasmon resonance (SPR) band at 521 nm, while upon the addition of Pd, the SPR band vanishes, indicating the coverage of Au by Pd.^48^Figure 2d shows that the Au@Pd core@shell nanoparticles are well dispersed on the alumina surface. The HAADF–STEM image in Figure 2e and the EDS line scan profile in Figure 2f further demonstrate the existence of the core@shell structure, showing that the core@shell structure remains during and after the deposition process. The orange line across the nanoparticle surface represents the scanning path with the direction from left to right. Moving along the scanning path, the counts for Pd start first, while the counts for Au are still at zero, indicating the probing of the ultrathin Pd shell. Scanning further, the counts for Au clearly increase, whereas the Pd counts are more or less constant. This indicates the probing of the Au core, which, of course, passes a maximum when the center of the spherical particles is scanned.

**Figure 3:**
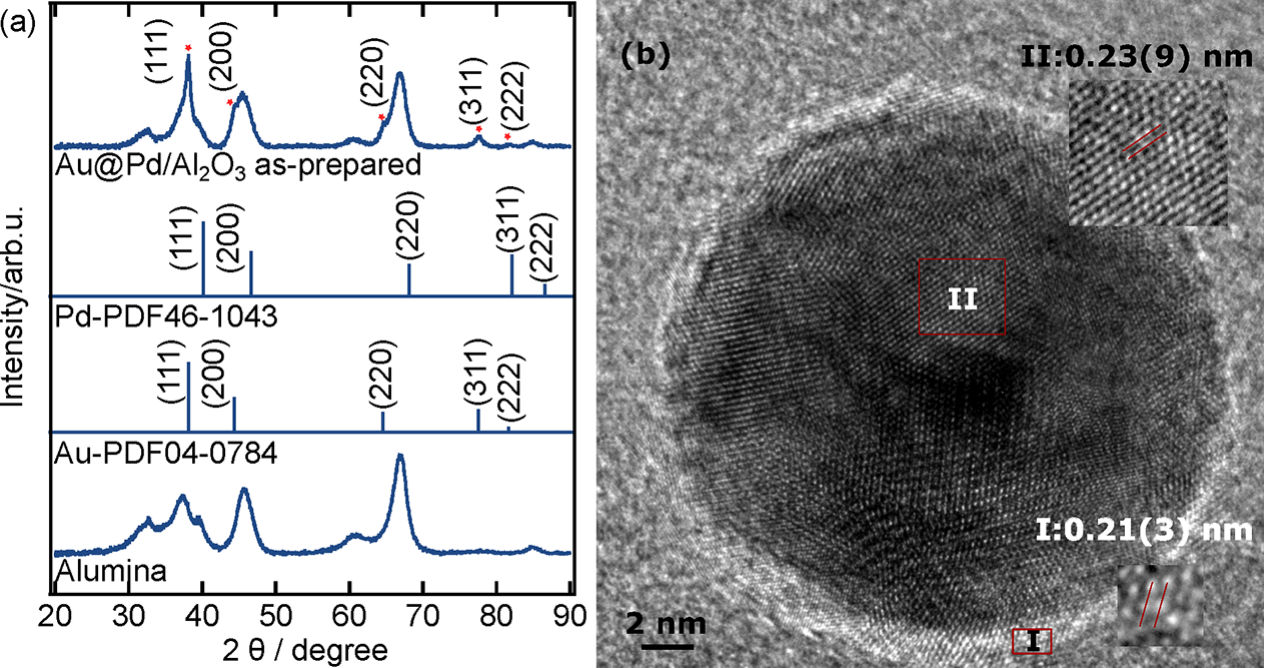
(a) XRD pattern of as-prepared Au@Pd/Al_2_O_3_ nanoparticles. (b) High-resolution TEM image of Au@Pd nanoparticles with measured lattice distance.

Turning to the crystal structure, powder XRD was used to collect the diffraction patterns, as shown in Figure 3a. The predominating peaks for the as-prepared Au@Pd/Al_2_O_3_ catalyst are at 2θ = 38.14, 44.63, 64.72, 77.59, and 81.82°. These are characteristic peaks for Au. Upon comparing the XRD patterns of as-prepared Au@Pd/Al_2_O_3_ with reference Au and γ-alumina, it is clear that the as-prepared Au@Pd/Al_2_O_3_ nanoparticles consist of highly crystalline Au cores with the fcc structure. Further analysis of the Au(111) reflection was performed after subtracting the pattern of pure alumina using the Scherrer equation, which gives an Au particle size of around 12 nm. This is in fairly good agreement with the analysis of the TEM images. This indicates that the deposition process of Au@Pd core@shell nanoparticles onto the alumina support proceeds without destroying the crystal structure. Further, comparing the XRD patterns of as-prepared Au@Pd/Al_2_O_3_ with reference Pd, it turns out that no Pd reflections could be identified. A likely reason for this is that the amount of Pd is too small to give rise to detectable scattering, and/or the Pd shell is too thin.^49,50^ Another reason could be that Pd peaks are overlapped by reflections from the alumina. Nevertheless, the absence of Pd peaks should not be mistaken for an amorphous Pd shell. On the contrary, the high-resolution TEM image shown in Figure 3b clearly reveals the existence of an ordered crystal structure in the Pd shell. The measured lattice distance for one selected area in the Pd shell is 0.21(3) nm, which could be ascribed to the lattice distance of Pd(111) planes.^49^

### Morphology and Crystallinity of the Used Catalyst

As discussed above, the anchoring of prefabricated nanoparticles to a support upon deposition is often weaker compared to when the support is impregnated with the active element(s). This may lead to severe sintering of nanoparticles forming larger agglomerates when used as a catalyst at elevated temperatures. Targeting low-temperature applications, that is, catalytic reactions below 250 °C, sintering issues are likely negligible. Nevertheless, the core@shell structure may be destroyed by catalytic side reactions when exposed to reaction conditions. This would inevitably change the arrangement of the shell surface atoms altering their catalytic properties. It is thus of great interest to study the structure of the Au@Pd/Al_2_O_3_ catalyst after being exposed to catalytic experimental conditions to get, at least, a first impression of its structural stability. Here, we characterized the morphology, crystallinity, and particle surface composition of the Au@Pd/Al_2_O_3_ catalyst after being used for CO oxidation at temperatures up to ca 200 °C (experiments that are discussed further below).

**Figure 4:**
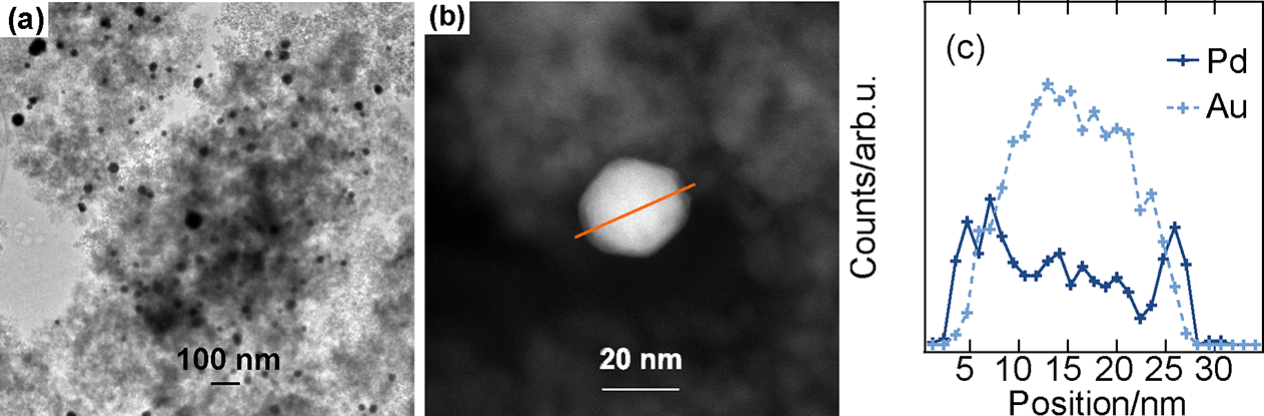
TEM (a) and HAADF–STEM (b) images and EDS line scan (c) of a Au@Pd/Al_2_O_3_ catalyst after being used for CO oxidation. The nanoparticles are well dispersed with the preserved core@shell structure.

Again, TEM and HAADF–STEM imaging and EDS line scan profiling were used to investigate the morphology of the used Au@Pd/Al_2_O_3_ catalyst as seen in Figure 4a–c, respectively. The TEM image reveals that the Au@Pd nanoparticles are still well dispersed on the alumina support. No significant sintering or agglomeration can be observed, suggesting minor morphological changes upon CO oxidation. The HAADF image and the EDS line scan profile across the nanoparticles further show that the core@shell structure in the supported Au@Pd nanoparticles is well preserved.

**Figure 5:**
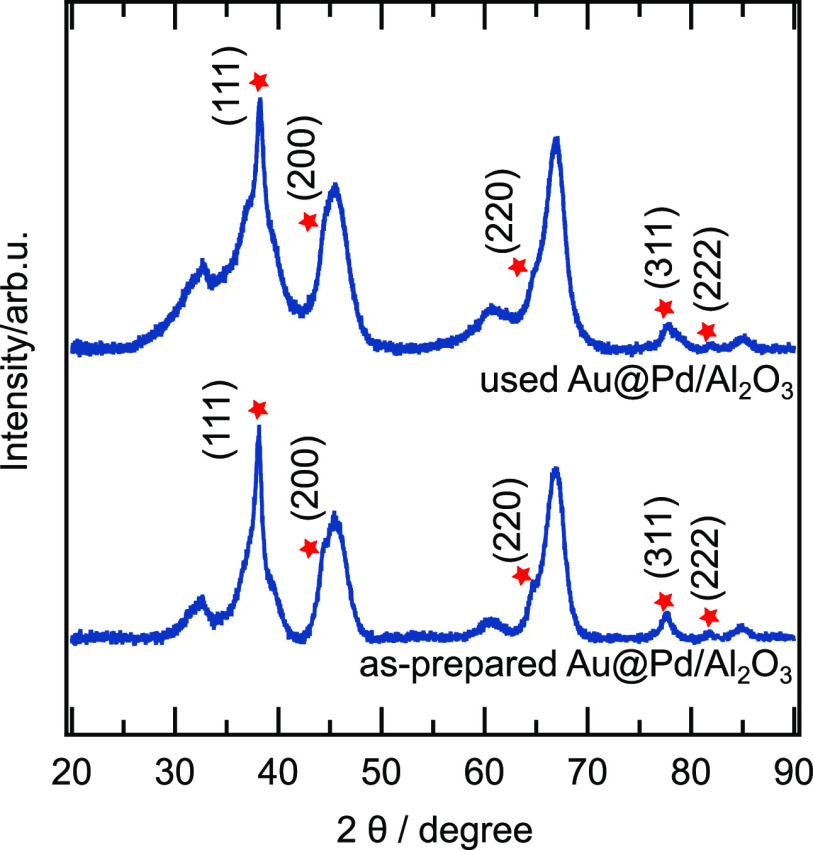
XRD patterns of the used and as-prepared Au@Pd/Al_2_O_3_ catalyst.

Concerning crystallinity, the used Au@Pd/Al_2_O_3_ catalyst was characterized with powder XRD. Figure 5 shows the resulting XRD pattern together with the corresponding pattern for the as-prepared Au@Pd/Al_2_O_3_ as a comparison. In essence, the two patterns resemble each other showing the same reflections, which suggests the fcc structure of the Au core. Furthermore, no reflections from Pd or PdO can be observed suggesting that the Pd shell is still intact, that is, no formation of crystalline Pd or PdO particles. Whether or not any “mixing” of Au and Pd occurs at the core–shell interface, which would induce a shift of the Au peaks, is difficult to say. Likely, the present instrumental resolution is too low to detect such shifts, especially considering it being an interface phenomenon only where just part of the already low Pd content may contribute. Further, no significant changes of the XPS spectra due to the reaction can be observed (cf. Figure S4), indicating a stable sample at these conditions.

### Surface Properties of the Pd Shell

CO is an often-used probe molecule in heterogeneous catalysis. Thanks to its selective adsorption on metal sites, the number of metal sites for a specific catalyst can be counted by measuring the CO uptake during CO chemisorption. This number constitutes a common basis for determining the metal dispersion, the average particle size, and the normalization of reaction rates. Further, when combined with IR spectroscopic characterization, CO chemisorption can be used to get structural information about both the adsorption layer and the catalyst site(s) through the vibration frequencies of adsorbed CO species.^51,52^ Thanks to the CO bonding mechanism, which involves back donation of electrons from Pd to bonded CO, the vibration of the C–O bond changes, and thus, the corresponding IR band carries information about the adsorption configuration and electronic properties of that site. The C–O vibrations are also sensitive to lattice effects such as strain. Hence, for the present Au@Pd core@shell system, the surface properties of the shell as compared to oxide-supported monometallic Pd nanoparticles can potentially be identified through the vibration frequencies of the CO ad-species.^53,54^ Specifically, adsorption of CO on palladium surfaces as well as particles results in linear, bridge, and hollow-bonded CO ad-species.^12,23,55−58^ Their relative abundance depends on the CO coverage, which for a given system is a function of temperature and CO pressure. It also depends on the surface structure, and for Pd particles, it is a function of the size and morphology and the type of support material.^22,59^ For small Pd particles and clusters supported on oxides, ionic Pd species can exist, which can bind CO in a linear configuration. However, the resulting IR band is considerably blue-shifted as compared to linear CO ad-species on a surface.^60^ Further, the positions of the IR bands depend on the CO coverage. This is not only due to the bonding mechanism but also due to the repulsive interaction between adsorbed CO species becoming increasingly significant with increasing CO coverage.

**Figure 6:**
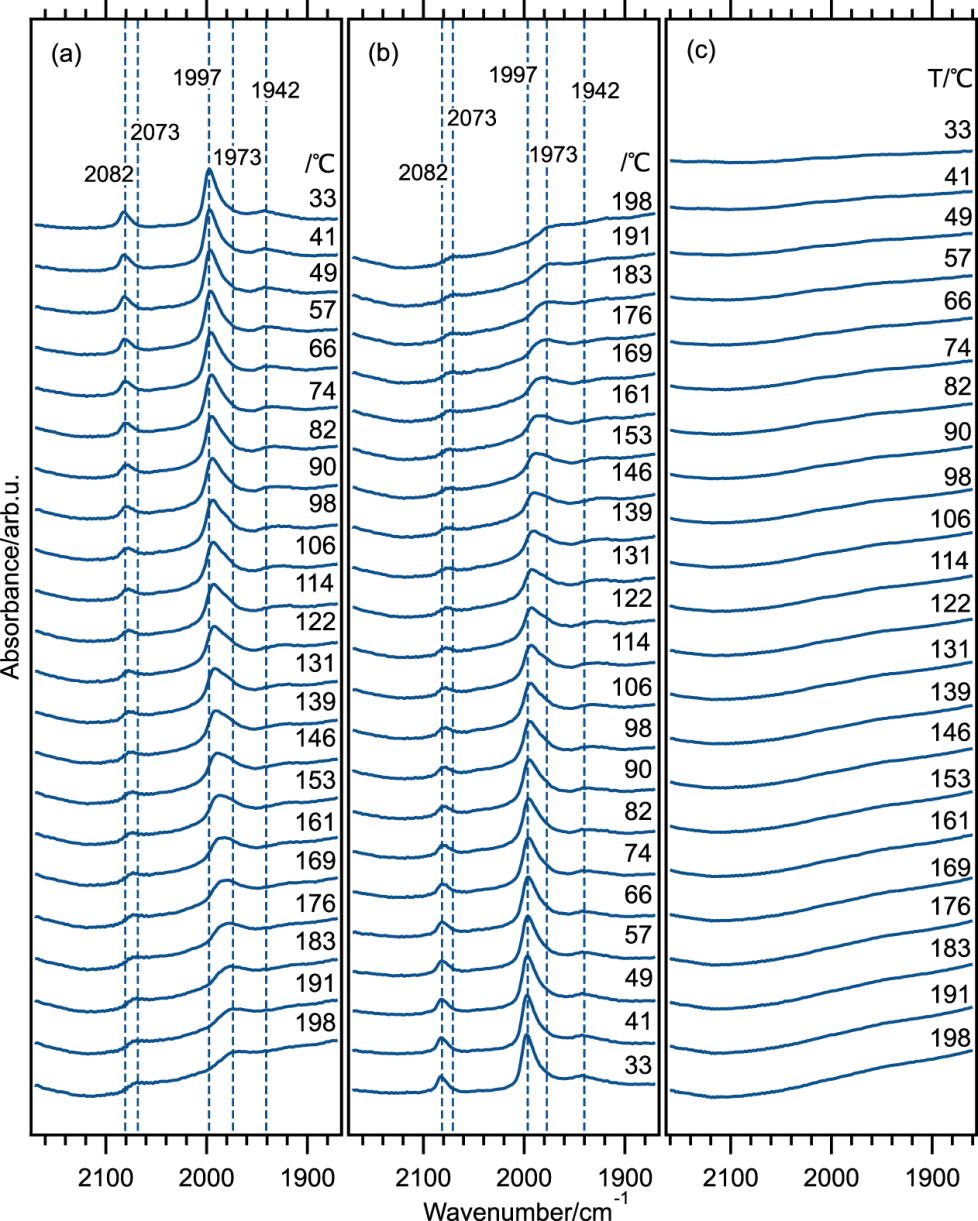
DRIFT spectra of (a) adsorption and (b) desorption of CO for as-prepared Au@Pd/Al_2_O_3_ with 1% metal loading. (c) Adsorption of 0.2% CO over Au/Al_2_O_3_ at different temperatures.

Figure 6a,b shows the IR spectra for the as-prepared Au@Pd/Al_2_O_3_ during the adsorption and desorption of CO, respectively. Figure 6c shows the spectra for adsorption of CO on the Au/Al_2_O_3_ for reference. In these spectra, the contribution from gaseous CO has been subtracted in order to display the IR bands of the CO ad-species of interest more clearly. This subtraction is necessary because the total number of surface sites in the present sample is low, making the analysis generally challenging. As can be seen, absorption bands for CO ad-species can only be observed for the Au@Pd/Al_2_O_3_ sample. The band at 2073 cm^–1^ that shifts to 2082 cm^–1^ with decreased temperature is assigned to CO linearly bonded on Pd and denoted with  (Pd). The bands at around 1942 and 1973 cm^–1^, shifting to 1997 cm^–1^ with decreasing temperature, are assigned to bridge-bonded CO on Pd and denoted with  (Pd). The frequency shifts and final positions of these IR bands at 33 °C are in good agreement with previous studies on CO adsorption on low-index Pd surfaces and especially Pd(111).^55,56,61^ As mentioned, the positions of the IR bands for CO ad-species are expected to shift with changing CO equilibrium coverage such that with increasing coverage, the repulsive CO adsorbate–adsorbate interactions become increasingly significant. Wei et al.^62^ also suggested that a more ordered adsorbate structure will form at low temperatures based on measurements of CO adsorption on a Pd film. They observed not only a blueshift but also a sharpening of the  (Pd) upon cooling the system from 450 to 80 K. The present results are qualitatively in line with these observations. Further, the two IR bands at higher wavenumbers are well in line with results for CO adsorption on silica-supported Pd particles, which were considered to be nearly nonaffected by the silica support and expose a significant proportion of Pd(111) facets.^57^ In terms of site abundance, the present results suggest that the surface of the Pd shell resembles that of the Pd(111) surface. Likely, the Pd shell surface exposes mostly Pd(111) facets with similar surface properties as the Pd(111) single crystal. Although the Pd shell is thin, the Au core seems to have negligible influence on the Pd shell as compared to the Pd single crystal concerning CO adsorption on exposed surface sites.

Considering the evolution of the IR bands as a function of temperature, it is clear that as the temperature is decreased, the  (Pd) band is the one observed first in the shape of a small and rather broad feature at 191 °C. As the temperature is decreased further, this band grows and becomes rather sharp at 33 °C. The  (Pd) may be seen at 191 °C, but a clear growth can be seen first when the temperature reaches 176 °C. The  (Pd) band at 1942 cm^–1^ can be seen only below 82 °C in our system. The weak intensity of  (Pd) suggests a relatively low abundance of this species,^63^ although one should be careful with concluding this as the attenuation coefficient (usually) is different for different CO ad-species (this has recently been discussed for supported platinum particles^64^). The accumulation of bridge-bonded CO species starting at higher temperatures as compared to the linear ones reveals that CO is preferentially adsorbed on the Pd surface in bridge-bonded configuration on these nanoparticles.

In order to further rationalize and compare the different IR bands obtained by experiments, the calculated frequencies for ontop, bridge, hcp, and fcc adsorbed CO as obtained by density functional calculations are shown in Table 1. Although there is a notable difference between the measured and calculated frequencies, there is a clear classification of the frequencies. The ontop (linear) CO has a frequency ranging between 2047 and 2144 cm^–1^, while the frequency of the bridging CO ranges between 1867 and 1982 cm^–1^ depending on the coverage. As for the fcc and hcp sites, the frequencies are very similar and range between 1792 and 1930 cm^–1^. The calculated frequencies agree well with what has been reported in the literature.^65^ The calculated results support the experimental observations of ontop and bridging configurations and a shift to higher frequencies at lower temperatures, that is, higher coverages.

**Table 1:** Symmetric Vibrational Frequency of CO at Different Coverages as Calculated in the *p*(2 × 2) Surface Cell^a^

site	0.25 ML	0.5 ML	0.75 ML
atop	2047	2066	2144
bridge	1867	1928	1982
hcp	1792	1878	1925
fcc	1795	1890	1930

a Units are in cm^–1^.

Compared to highly dispersed palladium catalysts synthesized with impregnation methods, the present sizes are an order of magnitude larger. Thus, the bridge-bonded CO species are expected to be dominating. This is in accordance with the previous study on CO uptake on Pd/Al_2_O_3_ catalysts with a systematic variation of Pd particle size reported by Velin et al.^22^ We mention that the spectra obtained for an Au@Pd/Al_2_O_3_ catalyst with lower nanoparticle loading (<1%) under the same experimental conditions are qualitatively similar (not shown). This strengthens that the observations are caused by the properties of the nanoparticles rather than being a matter of nanoparticle loading onto the alumina.

Compared with alumina-supported Pd particles, with typical sizes in the range of 2–10 nm, the IR bands for the CO ad-species generally appear at different wavenumbers for Au@Pd/Al_2_O_3_. Experimentally, we cannot unambiguously reveal whether or not this is caused by the Au core exerting a different effect on the palladium than the alumina or if it is due to varying morphology of the palladium phase, for example, the abundance of different sites and/or strain effects in the alumina-supported Pd particles. Comparing the CO adsorption IR spectra with the corresponding spectra for Al_2_O_3_-supported Pd only catalysts collected under identical experimental conditions, the  (Pd) band appears at a higher wavenumber, that is, 1997 instead of 1985 cm^–1^.^22^ One may speculate that when the Au core acts as a support for the Pd shell, charge is transferred from Pd to Au as the latter exhibits higher electronegativity than palladium.^66^ In response, this is expected to lead to a decreased electron back donation from Pd to the C–O bond^25,67^ blue-shifting the  (Pd) band. This is in part supported by the XPS measurements displayed in Figure S4 that also suggest a charge transfer from Pd to Au as the binding energy of Au 4f in Au@Pd/Al_2_O_3_ is shifted by around 0.7 eV to a lower value compared to pure Au. However, this could also be interpreted as an alloying between the Au core and the Pd shell at the very interface neither visible in XRD measurements nor influencing the CO adsorption on the Pd shell surface. Furthermore, caution should be taken when making comparisons between different supports. Although the support material clearly is different, the palladium particle size distribution is also different. For example, for alumina-supported palladium, the Pd particles are smaller, and a different family of surface sites with different coordinations cannot be ruled out, which would affect the IR bands. Further, the smaller Pd particles are most likely more strained, which could explain their red-shifted IR bands as compared to Au@Pd/Al_2_O_3_. This is indirectly supported by DFT calculations (not shown) that show a smaller impact of charge transfer than the strain on the CO vibration frequencies. This motivates further in situ and theoretical studies on core@shell systems with even thinner shells and varying core sizes to disentangle the effects of the support and strain.

### In Situ DRIFTS Characterization of Prototypical CO Oxidation

**Figure 7:**
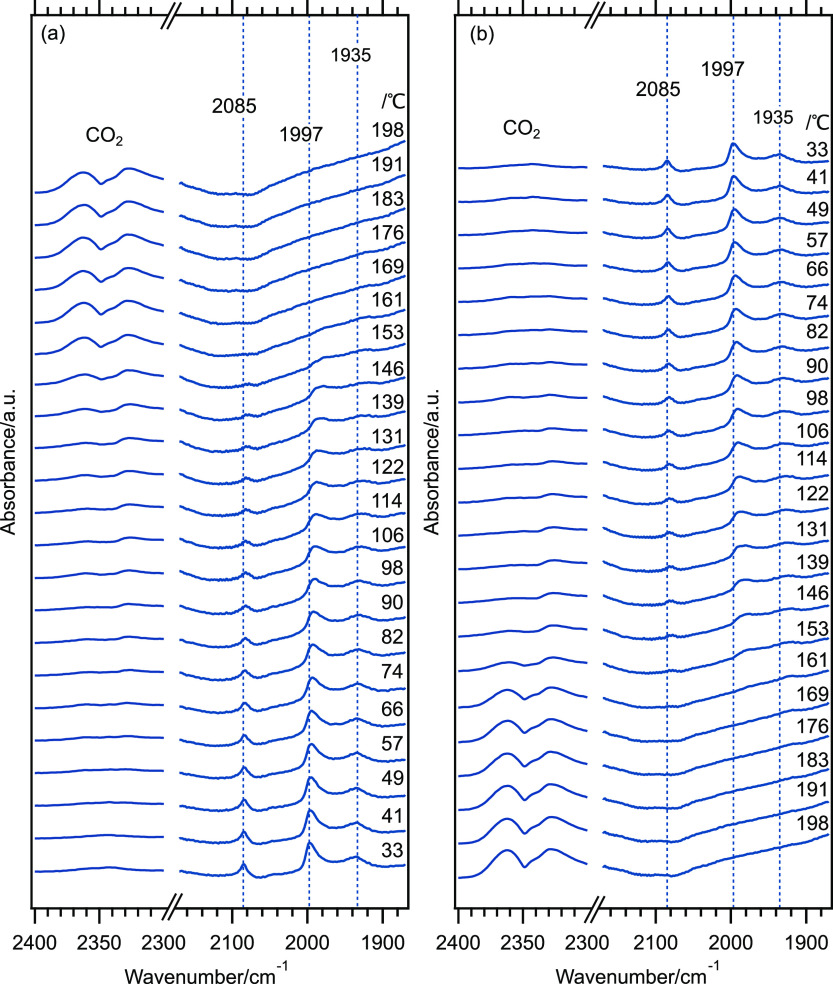
DRIFTS spectra for CO oxidation during (a) ignition increasing the temperature from 33 to 198 °C and (b) extinction decreasing the temperature from 198 to 33 °C over as-prepared Au@Pd/Al_2_O_3_ nanoparticles. The feed CO concentration is 0.2%.

The catalytic oxidation of CO is one of the most studied reactions in heterogeneous catalysis because of its important roles in applications but also thanks to its (apparent) simplicity making it a useful prototypical reaction for catalyst research and development.^12^ The common route for CO oxidation over palladium includes three steps, that is, associative CO adsorption, dissociative O_2_ adsorption, and surface reaction between adsorbed CO and O, in a Langmuir–Hinshelwood type of mechanism^68^ to form CO_2_ that immediately desorbs, viz.
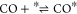

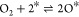

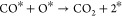
where * denotes an active site. The adsorption processes are competitive, favoring CO adsorption at low temperatures, and as the surface reaction is rapid, the reaction exhibits so-called kinetic phase transitions. These transitions occur when passing through critical conditions, for example, temperature and/or pressure, and lead to a drastic change in the adsorbate composition and, consequently, a drastic change in the reaction rate. At relatively low temperatures/high CO concentrations, the surface is nearly completely covered with CO and the reaction rate is low (CO self-poisoning), whereas at relatively high temperatures/low CO concentrations, O_2_ adsorption is appreciable, leading to high reaction rates. Expressing the reaction rate as a function of temperature (or CO/O_2_ concentration), the high and low reaction rate branches overlap, creating so-called bistability. At realistic conditions, the bistable kinetics, especially the transient kinetics, is complicated by the formation/removal of PdO. As mentioned in the Introduction, PdO may develop and expose under-coordinated Pd atoms exhibiting superior oxidation activity. However, the full structural characterization of the Pd shell surface is beyond the present scope. Instead, we used in situ DRIFTS to study the adsorbate composition during ignition/extinction of the CO oxidation over the Au@Pd/Al_2_O_3_ catalyst upon increasing/decreasing the temperature in the interval 33–198 °C. As mentioned, the indicated temperature is valid for the top layer of the catalytic bed, that is, where the IR probing occurs, but not representative of the entire bed.^69^ The collected DRIFTS spectra are shown in Figure 7a,b for the ignition and extinction processes, respectively. Again, the contribution of CO in the gas phase has been subtracted from the spectra.

Starting off with the CO ignition spectra and following the assignments from above, the  (Pd) and  (Pd) bands are clearly seen from the start at 33 °C. The  (Pd) and  (Pd) bands signify CO-covered Pd shell surfaces and that the catalyst operates in the CO self-poisoned regime as supported by the negligible formation of product CO_2_ (the double band centered at around 2348 cm^–1^). Upon increasing the temperature, the  (Pd) and  (Pd) bands become less intense, which reflects decreasing CO coverage. At around 100 °C, the IR band intensities of all CO ad-species have clearly decreased and some CO_2_ formation can be discerned. The lower CO coverage gives room for dissociation of O_2_ and further reaction with adsorbing CO and thus the onset of the CO oxidation reaction. At around 150 °C, the intensities of the IR bands for all ad-species are very low, signifying (negligible) low CO coverage. Here, the formation of CO_2_ is rather clear. From here and on, a sufficient number of free sites are available for oxygen dissociation and further reaction with adsorbing CO. Due to the rapid reaction, any CO molecule that adsorbs immediately reacts away at these conditions. Hence, upon increasing the temperature further, the only IR band that change is the one for product CO_2_, which increases with increasing temperature. For the CO oxidation extinction measurements, the  (Pd) and  (Pd) bands are in essence the same and more or less mirrors the ignition experiment. Thus, the common kinetic bistability (ignition-extinction hysteresis) is not well pronounced at the conditions used here. The temperature required for the CO oxidation to start and produce CO_2_ appears to be slightly high. In this connection, one should take into account that the DRIFT setup is afflicted with disadvantageous temperature distribution, making precise kinetic studies generally difficult. Further, taking into account the low active surface area of the catalyst due to the low particle loading, the results are promising and should not be used to disregard further studies of the Au@Pd/Al_2_O_3_ system.

Finally, it is interesting to compare the positions of the CO IR absorption bands during the CO oxidation experiment with the corresponding bands during CO adsorption discussed above. Here, the  (Pd) bands are positioned at the same frequency. The  (Pd) band, however, appears slightly blue-shifted by approximately 3 cm^–1^ in the presence of the reaction mixture. This suggests that the  (Pd) band is more affected by oxygen on/in the Pd shell, which may lead to charge transfer from Pd to these oxygens and less transfer to adsorbed CO making the C–O bond stronger.

## Conclusions

This study shows that a two-step seeded-growth method can be used to synthesize Au@Pd core@shell nanoparticles with a thin (1.5 nm) Pd shell independent of the Au core size, provided the synthesis parameters, that is, temperature and Au/Pd molar ratio, are carefully controlled. The preformed nanoparticles can be deposited onto high surface area alumina to form a functional Au@Pd/Al_2_O_3_ core@shell catalyst. High-resolution transmission electron microscopy and HAADF–STEM imaging and EDS line scan profiling show that the core@shell structure is maintained in the loading process and also after being exposed to CO oxidation reaction conditions up to about 200 °C. IR characterization of CO adsorption and prototypical CO oxidation reveals that the Pd shell surface mainly exposes Pd(111) facets onto which CO can bind in known linear, bridged, and likely also hollow positions. The observed IR bands for the CO ad-species are blue-shifted by about 7 cm^–1^ as compared to alumina-supported Pd monometallic particles of sizes 2–10 nm, which is possibly explained by the smaller size of the latter including also more strain. Under reaction conditions, the positions of the IR bands for bridge-bonded CO remain, whereas the position of the IR band for linearly bonded CO blueshifts by approximately 3 cm^–1^. This suggests that the linear-bonded CO is more affected by the presence of oxygen on/in the Pd surface under reaction conditions. Finally, one may envisage that the core@shell motif provides opportunities to study the effect of electronic promotion of the shell by the core and strain in the shell if the size and thickness of the core and shell, respectively, are systematically varied.

## Supporting Information Available

Size distribution of Au nanoparticles; thickness distribution of Pd shells; UV–vis spectra of Au and Au@Pd particles; and XPS spectra of the Au@Pd/Al_2_O_3_ as-prepared sample and after catalytic DRIFTS characterization (PDF)
