# Supplementary material for: Synthesis and Characterization of Catalytically Active Au Core—Pd Shell Nanoparticles Supported on Alumina
Source: Langmuir. 2022 Oct 12;38(42):12859–70. doi: 10.1021/acs.langmuir.2c01834 (PMC9609311; doi:10.1021/acs.langmuir.2c01834)
Supplement: Supplementary file 1 — la2c01834_si_001.pdf [file la2c01834_si_001.pdf]

Supporting Information (SI)
Synthesis and characterization of catalytically active Au core - Pd shell nanoparticles supported on alumina

Yanyue Feng[a], Andreas Schaefer[a], Anders Hellman[b], Mengqiao Di[a], Hanna Härelind[a], Matthias Bauer[c] and Per-Anders Carlsson[a,*]

[a]Department of Chemistry and Chemical Engineering, Chalmers University of Technology, SE-41296 Gotheburg, Sweden

[b]Department of Physics, Chalmers University of Technology, SE-41296 Gothenburg, Sweden

[c]Department of Chemistry, Paderborn University, 33098 Paderborn, Germany

September 30, 2022

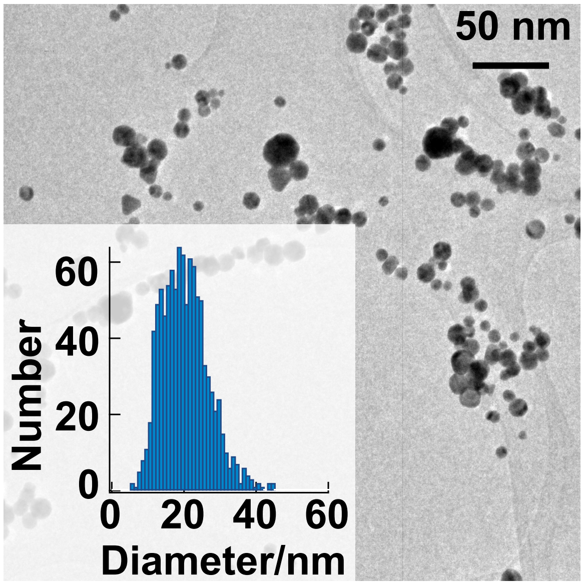

Figure S1: Size distribution of Au nanoparticles

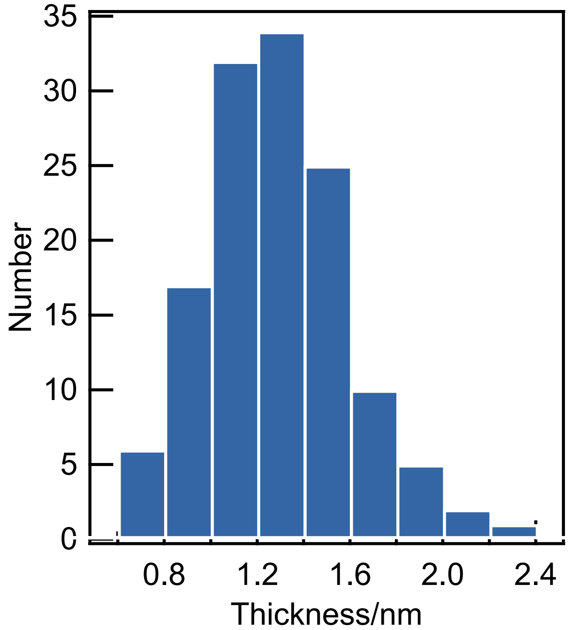

Figure S2: Thickness distribution of Pd shell for Au@Pd nanoparticles

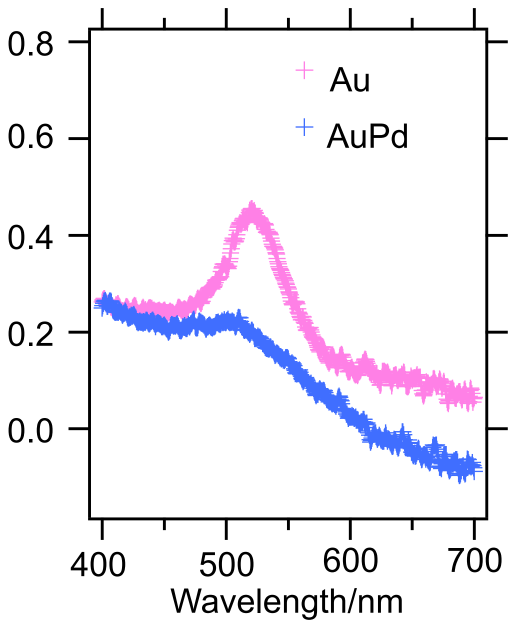

Figure S3: UV-vis spectra of Au and Au@Pd nanoparticles

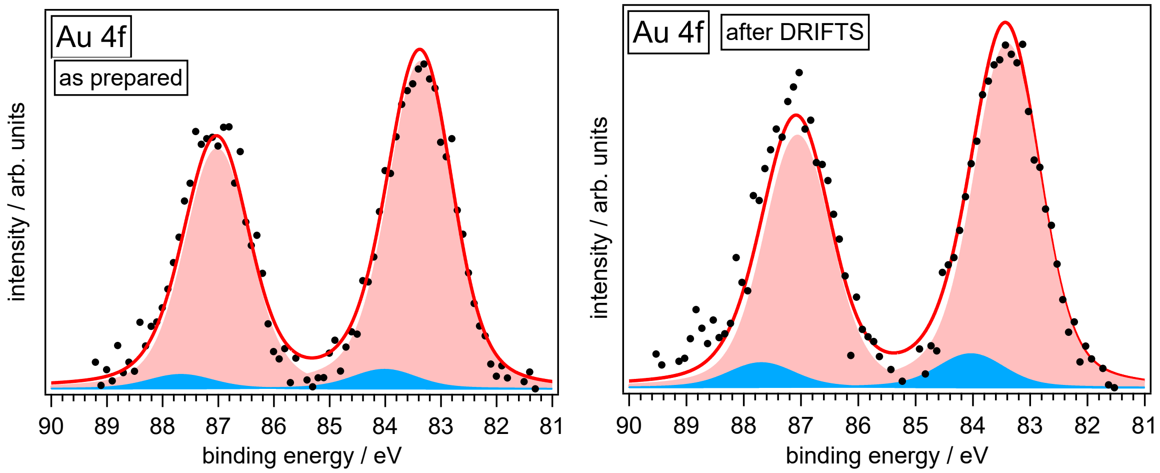

Figure S4: XPS spectra of Au 4f for Au@Pd/Al_2O_3 of the as prepared sample and after catalytic DRIFTS characterization

The Au 4f photoelectron spectra of 1 w% Au@Pd/Al_2O_3 of the as prepared samples and after a DRIFTS experiment are shown in fig. S4. The binding energy scale was calibrated to the position of the Al 2p signal at 74.4 eV which was determined by peak fitting. Due

to the low signal intensity a linear background was subtracted from the Au 4f spectra. The spectra were fitted with a Doniach-Sunjic (DS) line convoluted with a Gaussian. The width of the DS lines was fixed following [1]. The DS lines were further given a slight asymmetry following [2] assuming bulk metallic behavior. The spin orbit split was fixed to 3.67 eV [3]. The best fit was obtained for two signal pairs with the 7/2 components centered at 84 eV (bulk Au value) and 83.3±0.2 eV, respectively. A contribution expected from Pd 4s photoelectrons at 88-89 eV was deemed too small to attempt to include in the fit. The inelastic mean free path of a Au 4f photoelectron travelling through Pd metal would be about 18 Å. This means it is safe to assume a Au4f signal from the Au core of the particle to still be present in the 4f signal. Hence we incorporated such a component at 84 eV in the attempted deconvolution. The main Au4f signal is shifted by about 0.7±0.2 eV towards lower binding energy compared to bulk Au. This is a similar value as observed for Au and AuPd particles in TiO_2 [4]. At this point we can only speculate on the exact origin of the observed shift and do not exclude formation of an AuPd alloy boundary region between Au core and Pd shell. The binding energy calibration with respect to the insulating support is challenging and a more detailed study was beyond the scope of the measurements reported in this work. In essence, however, the results in figure S4 show that no significant difference is observed between the spectra recorded for the as-prepared sample and the sample after a DRIFTS characterization, indicating a stable sample for these conditions.

[1] J. L. Campbell and T. Papp, Widths of the atomic K-N7 levels. *Atomic Data and Nuclear Data Tables* **77**(1) (2001) 1–56.

[2] S. Hüfner and G. K. Wertheim, *Core-line asymmetries in the X-ray-photoemission spectra of metals*, Phys. Rev. B **11**(2) (1975) 678.

[3] J. F. Moulder, W. F. Stickle, P. E. Sobol and K. D. Bomben, *Handbook of X-ray photoelectron spectroscopy*, Perkin-Elmer Corp., Eden Prairie, MN, 1992.

[4] L. Delannoy *et al.*, *Surface Segregation of Pd from TiO_2-Supported AuPd Nanoalloys under CO Oxidation Conditions Observed In Situ by ETEM and DRIFTS*, ChemCatChem **5** (2013) 2707-2716.
